# The Combination of HSP90 Inhibitors and Selumetinib Reinforces the Inhibitory Effects on Plexiform Neurofibromas

**DOI:** 10.3390/cancers17142359

**Published:** 2025-07-16

**Authors:** Sajjad Khan, Oluwatosin Aina, Ximei Veneklasen, Hannah Edens, Donia Alson, Li Sun, Huda Zayed, Kimani Njoya, Daochun Sun

**Affiliations:** 1 Department of Cell Biology, Neurobiology and Anatomy, Medical College of Wisconsin, Milwaukee, WI 53226, USA; sajkhan@mcw.edu (S.K.); hannah.edens@aah.org (H.E.); knjoya@mcw.edu (K.N.); 2 Department of Pediatrics, Medical College of Wisconsin, Milwaukee, WI 53226, USA; 3 Cancer Center, Medical College of Wisconsin, Milwaukee, WI 53226, USA; 4 Children’s Research Institute, Milwaukee, WI 53226, USA

**Keywords:** NF1, plexiform neurofibromas, selumetinib, HSP90 inhibitors

## Abstract

**Background/Objectives**: Plexiform neurofibromas (pNFs) are one of the cardinal presentations of NF1 patients, often arising during early childhood. Since selumetinib was approved by the FDA in 2020, the long-term side effects and various responses of mitogen-activated protein kinase inhibitors (MEKi) in pediatric patients necessitate a new strategy. We propose that combining selumetinib with heat shock protein 90 inhibitors (HSP90i) can enhance the inhibitory effects as well as reduce the dosage of selumetinib in combination. We validated the synergistic effects and the significantly improved treatment effects of the combination of selumetinib and HSP90i in pNFs. **Methods**: We used drug screen data mining to predict the combination of selumetinib and HSP90i. Using cell lines and in vivo mouse models for pNFs, we tested a series of combinations with different concentrations. We validated the in vivo inhibitory effects using the transplanted tumors from *DhhCreNf1^f/f^* mouse models. **Results**: We demonstrated that combining selumetinib and SNX-2112 or retaspimycin can achieve better tumor inhibition with synergistic effects. The combination significantly delays the progression of mouse pNFs. **Conclusions**: The combination of selumetinib and HSP90i has significant synergistic effects, provides therapeutic inhibitor effects, and reduces the selumetinib dosage in combination.

## Simple Summary

Plexiform neurofibromas (pNFs) are common, benign tumors in pediatric patients with Neurofibromatosis type 1 (NF1). Selumetinib, an FDA-approved MEK inhibitor, raises concerns about its long-term side effects. Our drug screen data mining identified a list of heat shock protein 90 inhibitors that can be combined with selumetinib for improved therapeutic efficacy. We tested SNX-2112 or retaspimycin in combination with selumetinib and revealed the potent inhibition and synergistic effects in pNF cell lines. Furthermore, we demonstrated a better inhibitory effect of the combination of SNX-2112 and selumetinib on tumor growth in vivo, using the *DhhCreNf1^f/f^*-derived mouse pNFs.

## 1. Introduction

Plexiform neurofibroma (pNF) is one of the most clinically challenging manifestations of neurofibromatosis type 1 (NF1), a genetic disorder caused by the biallelic loss of the NF1 tumor suppressor gene. pNFs are characterized as benign peripheral nerve sheath tumors, but often cause significant morbidity due to their size, location, and tendency to infiltrate surrounding tissues. More critically, a subset of pNFs carries a high risk of progressing to malignant peripheral nerve sheath tumors (MPNSTs), which are highly aggressive and associated with poor prognosis [1,2]. Until recently, therapeutic options for pNFs remained limited, particularly for surgically inaccessible cases. The approval of selumetinib, a mitogen-activated protein kinase inhibitor (MEKi), for pediatric pNFs marked a significant therapeutic advancement in pNF treatment, representing the first FDA-approved pharmacological treatment for use in inoperable pNFs [3]. Selumetinib acts by targeting the hyperactive RAS/MAPK signaling pathway, which is commonly upregulated following loss of neurofibromin function in NF1-deficient cells. Despite clinical promise, selumetinib has demonstrated limited tumor responses, with variable clinical outcomes across patients, and long-term adverse effects in pediatric patients, including gastrointestinal, dermatological and cardiac side effects [4,5]. Reducing toxicity, enhancing the inhibitory effect, and preventing drug resistance are essential for the long-term clinical success of selumetinib and other MEKi, thereby maximizing patient benefit in a larger patient population.

Recent studies have highlighted intratumoral heterogeneity in NF1-associated tumors, particularly reflected by the stem-like tumor subpopulation, that may drive treatment resistance and recurrence in patients [6,7]. These stem-like tumor cells exhibit features similar to neural crest stem cells (NCSC), such as self-renewal, slower cell cycle rates and heightened plasticity, which enable them to adapt to new environments or resist standard treatments [7,8,9,10]. Notably, MEK inhibition has shown limited efficacy in eradicating stem-like tumor cells and may inadvertently promote stem-like characteristics by activating WNT or PI3K/AKT signaling pathways in various tumors [11,12]. These findings underscore the need for innovative therapeutic strategies to address these therapeutic challenges in pNFs by effectively suppressing all tumor subpopulations.

One emerging approach to address the inefficiencies of monotherapies involves employing combination therapies that target multiple pathways or molecular features of pNF [13]. A series of drug combination studies has demonstrated effectiveness in NF1-associated MPNSTs, including a combination of a SHP2 inhibitor and MEKi, which targets multiple vulnerabilities within the tumor [14,15]. While these studies predominantly focus on combining kinase inhibitors to interfere with aggressive growth, we rationalize targeting heterogeneous molecular features, especially stem-like features, through rationally selected drug combinations. Following this line of reasoning, we reanalyzed the drug screen data on pNFs to identify candidates that target stem-like regulatory networks and could be used in combination with selumetinib.

The drug screen data mining revealed that HSP90i are among the top-ranking drug candidates and are capable of targeting the stem-like gene regulation network [16]. HSP90 is a molecular chaperone essential for the stability and activity of a wide range of oncogenic proteins, including kinases and transcription factors involved in differentiation, stress response, and survival, and HSP90i have emerged as a promising class of agents in oncology [17]. Notably, the HSP90 family has been reported to be involved in stem cell differentiation [18], and HSP90 inhibition has been shown to suppress stem-like features in tumor models [19]. HSP90i has significant preclinical efficacy against various cancers, and over 22 HSP90i compounds have progressed into clinical trials [20]. Furthermore, the combination of HSP90i with various chemotherapeutics, immunotherapies, and targeted treatment has shown enhanced efficacy and reduced systemic adverse effects compared to monotherapy protocols [21]. Currently, there are 18 active clinical trials investigating HSP90 inhibitors in conjunction with other agents, highlighting a trend toward combinatory experimentation [20]. While HSP90i enhances the sensitivity of tumor cells to MEKi by inhibiting compensatory signaling mechanisms [22,23], its application has rarely been extended to targeting NF1-associated pNFs. Among the HSP90i list we analyzed, SNX-2112 and retaspimycin have shown remarkable efficacy in preclinical studies by destabilizing key oncogenic proteins involved in pNF growth and maintenance [24,25,26].

These findings highlight the intriguing prospect of combining HSP90i and selumetinib, which could potentiate the anti-tumor effects of selumetinib monotherapy. The novelty of this approach lies in its potential to overcome the limitations of selumetinib, reduce the dosage, and interfere with heterogeneous tumor molecular features.

## 2. Materials and Methods

### 2.1. Drug Screen Analysis and Combination Prediction

Drug screen data and pNF cell lines expression data were downloaded from the NFdata portal [27]. The conserved gene regulation networks and the correlation between drug responses and the gene network were determined using the WGCNA package v1.69, as described previously [16,28]. The IDACombo package was used to predict the combination efficacy using the drug screen data with the default settings [29].

### 2.2. Cell Culture and Viability Assay

pNF cell lines, ipNF95.11bC, ipnNF95.11c, ipNF05.5 Single Clone, and ipNF05.5 Mixed Clones were obtained from the American Type Culture Collection (ATCC, Manassas, VA, USA). Cells were cultured in Dulbecco’s Modified Eagle Medium (DMEM; Ref: 10566–016; Thermo Fisher Scientific, Waltham, MA, USA) supplemented with 10% fetal bovine serum (FBS; Ref: 978068–085; Avantor Sciences Central, Radnor, PA, USA) and 1% Antibiotic-Antimycotic (Anti-Anti; Ref: 45240–062; Thermo Fisher Scientific, Waltham, MA, USA). Cultures were maintained at 37 °C in a humidified incubator with 5% CO_2_. For combination drug testing, cells at ~80% confluence were seeded into Corning^®^ 96-well Flat Clear Bottom White Microplates (Ref: 3610; Corning Inc., Corning, NY, USA) and allowed to adhere to the 96-well plate overnight. The medium was replaced the next day with serum-free DMEM containing various concentrations of selumetinib (0, 6, 12, and 24 µM) systematically combined with either SNX-2112 (0, 0.047, 0.07, 0.105, and 0.1575 µM) or retaspimycin (0, 0.08, 0.16, 0.32, and 0.64 µM) in a matrix. Cells were incubated with the treatment for 48 h, followed by cell viability analyses. Cell viability was assessed using the CellTiter-Glo^®^ Luminescent Cell Viability Assay (Cat# G755B/G756B; Promega, Madison, WI, USA), according to the manufacturer’s instructions. Luminescence was measured at 573 nm using a microplate reader, and viability was expressed as a percentage of vehicle-treated controls. Drug synergy was evaluated using the Bliss independence model via the SynergyFinder online platform (https://synergyfinder.org/ (accessed on 7 July 2025).

### 2.3. In Vivo Drug Test

All animal procedures were conducted in accordance with protocol #7457, approved by the Institutional Animal Care and Use Committee of the Medical College of Wisconsin. pNF tumors were generated in nude mice by transplanting tumor cells harvested from dorsal root ganglia with *DhhCre;Nf1^f/f^;Luciferase* configuration. Following harvest, tumors were enzymatically dissociated into a single-cell suspension, and 5 × 10^4^ cells were injected into the right sciatic nerve of 6-week-old female nude mice. After confirming tumor engraftment using an in vivo imaging system (IVIS), mice were randomly assigned into three treatment cohorts (*n* = 5 per group): (1) vehicle control, (2) selumetinib monotherapy (50 mg/kg/day, orally), and (3) combination therapy with selumetinib (40 mg/kg/day, orally) and SNX-2112 (30 mg/kg/day, intraperitoneally). Treatments were administered daily for 17 days. The animals were given painkillers before and after surgery according to the protocol. The specific vet team and experimenters observed the experimental mice regularly. At the study endpoint, all mice were euthanized, and tumors were harvested for subsequent analyses. Tumor growth was indicated by bioluminescence. The final sizes were measured by a caliper at Day 18 and calculated by ½ × length × width^2^.

### 2.4. Statistical Analyses

All graph generation and statistical analyses were conducted using GraphPad Prism (v10.4.0; GraphPad Software, San Diego, CA, USA). Results are presented as mean ± standard error of the mean (SEM) of three independent biological replicates. Statistical significance was determined by using one-way ANOVA to compare tumor volumes of treatment cohorts with the control. Two-way ANOVA was used to assess the statistical significance of various treatments over time relative to the control. A *p*-value of 0.05 or less was considered statistically significant.

## 3. Results

### 3.1. The Combination Targets Heterogeneous Molecular Features

**Figure 1:**
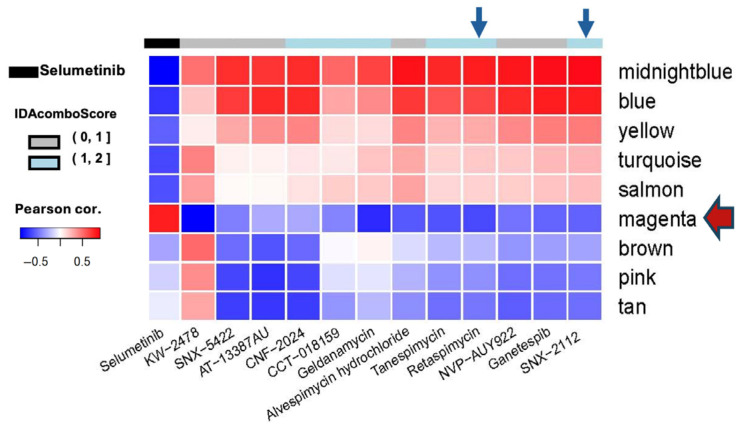
Correlation pattern between the drug responses and conserved gene regulation networks in pNF drug screen. Selumetinib is indicated in the first column, and all other HSP90i in MIPE4.0 were included. The IDAcombo scores are indicated by the color bar. Blue arrows indicate the columns of retaspimycin and SNX-2112. The red arrow indicates the magenta network.

Our previous characterization of conserved gene regulation networks in pNF cell lines and primary tumor tissues revealed a series of essential networks [16]. These conserve networks were defined by the WGCNA algorithms with a number of co-regulated genes within each color-named network. With the gene ontology analysis, we revealed the potential biological relevance with specific network, specifically the magenta reflecting the stem-like signatures [16]. The original pNF drug screen included six immortalized Schwann cell lines with different NF1 gene mutation statuses [30]. The ipNF95.11bC (NF1^-/−^) and ipnNF95.11c (NF1^+/−^) were immortalized from the same pNF patient using the tumor tissue and tumor-adjacent non-tumor Schwann cells, respectively. ipNF05.5 Mixed Clones (*NF1^−/−^*) and ipNF05.5 Single Clone (*NF1^−/−^*) were generated from the same pNF tumor, the former being a pool of 6 different clones derived from ipNF05.5 Single Clone. We reanalyzed the drug screen data that utilized the MIPE 4.0 library to screen drugs against those pNF cell lines [31]. Complementary patterns were revealed when we correlated the drug responses to different concentrations of selumetinib and HSP90i using the conserved gene regulation network, as shown in Figure 1. The responses to selumetinib negatively correlate with the gene expression of conserved gene networks, except for the magenta, indicating that the higher expression of stem-like signatures in magenta is concurrent with increased cell viability upon treatment with selumetinib. This result indicates that selumetinib may not target the stem-like molecular features represented by the magenta network. In contrast, most of the HSP90i are negatively correlated with the magenta network, implying that these drugs may target the stem-like molecular properties associated with this network. To evaluate the drug combination potential between HSP90i and selumetinib, we use the IDAcombo R package to calculate the IDAcombo Score, which predicts the combination benefit based on the individual effects of the two drugs [29]. The IDAcombo scores between HSP90i and selumetinib, as shown in Figure 1, are represented by the top color bar. We hypothesized that HSP90i can enhance the therapeutic efficacy of selumetinib and reduce the dosage of selumetinib in combination. We selected SNX-2112 and retaspimycin for further combination validation.

### 3.2. SNX-2112 and Retaspimycin Improve the Inhibitory Effect of Selumetinib

**Figure 2:**
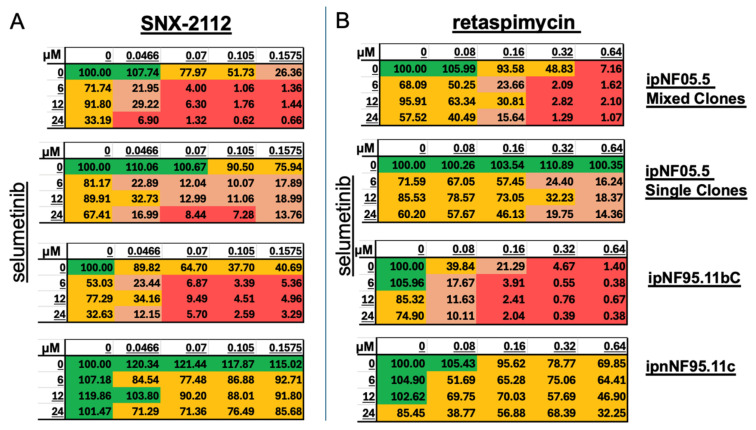
Effect of drug combinations on cell lines. Cell viability of pNF and Schwann cell lines with the treatment of (**A**) selumetinib and SNX-2112 or (**B**) selumetinib and retaspimycin. The concentrations of selumetinib are indicated as row names and concentrations of SNX-2112 or retaspimycin are indicated as column names. The mean viability is normalized to the controls with only vehicle treatment. *N* = 3.

We employed the ATP-based cell viability assay to assess the efficacy of the combination of selumetinib and SNX-2112 or retaspimycin at varying concentrations (Figure 2). The four cell lines, ipNF05.5 Mixed Clones, ipNF05.5 Single Clone, ipNF95.11bC, and ipnNF95.11c, were utilized to evaluate the effects of drug combinations. The concentrations of selumetinib (0, 6, 12, 24 µM) and SNX-2112 (0, 0.0466, 0.07, 0.105, 0.1575 µM) or retaspimycin (0, 0.08, 0.16, 0.32, 0.64 µM) were systematically combined in a matrix and evaluated in triplicate using 96-well plates. As illustrated in Figure 2A,B, treatment with selumetinib alone (the left column) resulted in modest dose-dependent responses in pNF cell lines. The administration of the lowest concentrations of HSP90i, 0.0466 µM of SNX-2112 or 0.08 µM of retaspimycin in combination, yielded significantly improved inhibition compared to selumentinib alone. Meanwhile, the combinations exhibited mild inhibitory effects on non-tumor Schwann cell lines, ipnNF95.11c. These findings indicate that the combinations of selumetinib and HSP90i effectively inhibit pNF cell lines. Furthermore, the combination of SNX-2112 dramatically reduces the dosage of selumetinib, achieving a higher level of inhibition or providing more efficient inhibition than selumetinib alone.

### 3.3. The Combinations Demonstrate Strong Synergistic Effects in Pnf Cell Lines

**Figure 3:**
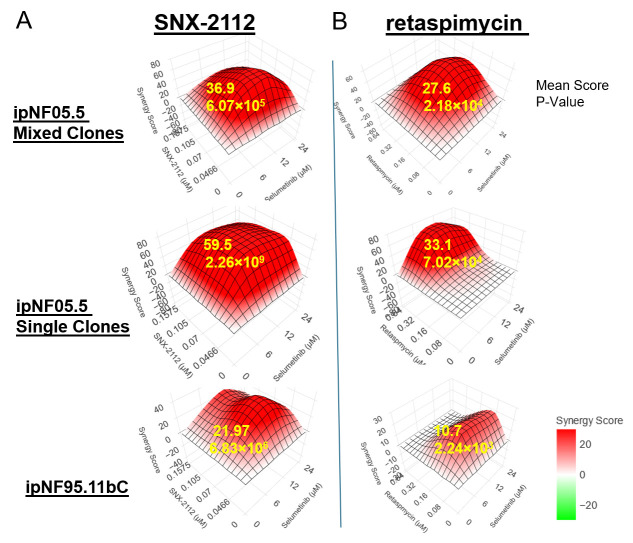
Bliss synergy analyses of combinations. (**A**) Selumetinib and SNX-2112 and (**B**) Selumetinb and retaspimycin. The Bliss synergy scores are calculated, and the mean score and *p*-value are calculated and indicated on the 3D heatmap generated by SynergyPlus.

Given the promising inhibitory effects of SNX-2112 and retaspimycin, we further assessed the synergistic effects between selumetinib and the two HSP90i. Utilizing the SynergyPlus web tool [32], we calculated the Bliss synergy score based on the viabilities of the cell lines treated with the combination matrix (Figure 3). The synergistic effect score cutoff defined by the SynergyPlus algorithm is that the mean synergy score should be more than 10 [33]. As illustrated in Figure 3A,B, the means of synergy scores for the ipNF05.5 Mixed Clones, ipNF05.5 Single Clone, and ipNF95.11bC are all above the cutoff for the combinations. Notably, the highest synergy scores were commonly observed within the combination therapy, even at low concentrations of both agents. The confirmation of synergistic effects in these NF1-deficient cell models further underscores the outperformance of the combination treatment over selumetinib alone.

### 3.4. The Combination Shrinks the Pnf More than Selumetinib

**Figure 4:**
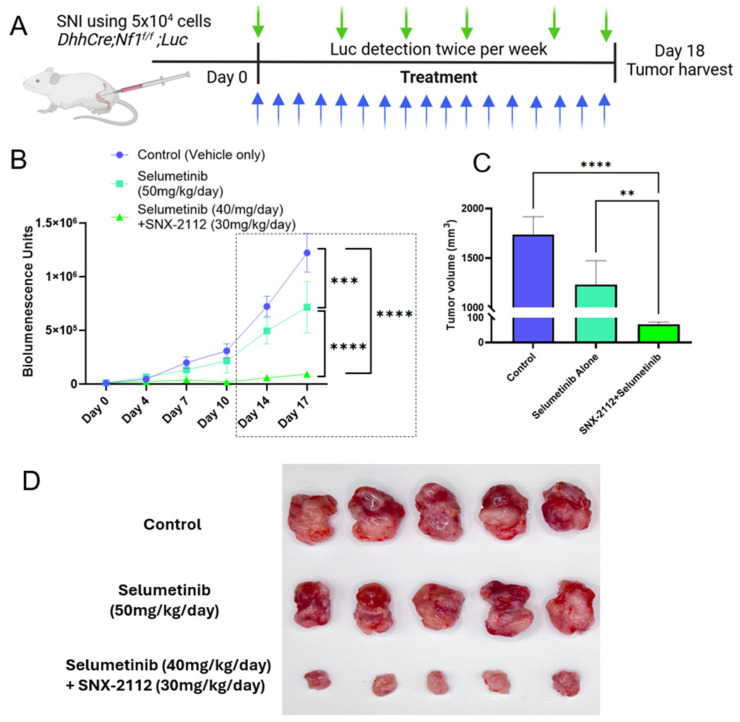
In vivo treatments on transplanted pNFs by sciatic nerve injection (SNI) using vehicle control, selumetinib, and combination. (**A**) Treatment scheme. Green arrows for luciferase (Luc) detection and blue arrows for treatment. (**B**) Tumor growth curves of different cohorts monitored by bioluminescence. Two-way ANOVA of bioluminescence demonstrates significant differences among the three cohorts at Day 14 and Day 17, *** *p* < 0.001, **** *p* < 0.0001. (**C**) Tumor volumes were determined by a caliper on Day 18. One-way ANOVA, ** *p* < 0.01, **** *p* < 0.0001, *N* = 5, Mean ± SEM. (**D**) Tumors were dissected from different cohorts on Day 18.

The therapeutic potential of the combination is further evaluated through the orthotopically transplanted mouse pNFs derived from the *DhhCreNf1^f/f^;Luciferase* model [34]. Fifteen transplanted pNFs were generated and then randomly assigned to three cohorts, which were treated with vehicle control, selumetinib (50 mg/kg), or the combination of selumetinib (40 mg/kg) and SNX-2112 (30 mg/kg). The daily treatment started three days after transplantation (Figure 4A). On Day 0, bioluminescence from the transplanted tumors was determined using the IVIS system, and the monitoring was repeated twice a week. In the combination cohort, we reduced the dosage of selumetinib by 20% compared to the selumetinib alone cohort. Also, we utilized a substantially lower dosage of SNX-2112 than what has been reported in prior studies [24,35,36], which enables us to challenge the hypothesis that the combination treatment may not only enhance its inhibitory effects in vivo but also diminish the dosage of selumetinib. Significant differences were revealed by the comparison of the three cohorts. The growth rate of tumors demonstrated a much stronger inhibitory effect of the combination compared to both selumetinib alone and vehicle control (Figure 4B) at different time points. The significantly different tumor sizes at the endpoint on Day 18 are shown in C based on caliper-measured sizes. The tumors of three cohorts are shown in Figure 4D, with no significant toxicity reflected by body weight loss.

## 4. Discussion

The heterogeneous molecular features of tumor cells may have various drug sensitivities, which rationalizes the need for combination treatment strategies. By leveraging synergistic effects that act on complementary molecular features, combination therapies can potentially circumvent the treatment challenges, such as partial response, drug resistance, and drug toxicities, especially associated with single drug usage in high doses. Keeping these factors in view, this study integrates drug mining analyses with in vitro and in vivo studies to identify and validate effective drug combinations for targeting pNFs. Our findings collectively underscore the promise of combining selumetinib, a MEK inhibitor and HSP90 inhibitors, SNX-2112 and retaspimycin. This combination strategy reinforces the inhibitory effects on pNF growth as well as offering the potential to reduce selumetinib toxicity by reducing its dose while enhancing efficacy.

MEK inhibition serves as a reasonable treatment for tumors driven by *NF1* gene mutations, which lead to hyperactivation of the RAS/MAPK signaling pathway. Selumetinib, a MEKi approved by the FDA in 2020 for use in pediatric patients with inoperable pNFs, has shown acceptable responses in patients, nonetheless, with variable clinical outcomes. Similarly, Mirdametinib, another MEKi, was recently approved by the FDA in 2025 for adult pNF patients, with a confirmed objective response rate of 41% in clinical studies [37]. This variation in clinical response highlights the limitation of MEKi monotherapy, which can be possibly attributed to intratumoral heterogeneity. In this study, it was observed that SNX-2112 and retaspimycin significantly overcame some of the limitations of selumetinib. HSP90i acts by destabilizing oncogenic substrates that are reliant on HSP90 chaperone activity for stability and activity. This includes receptors and signaling proteins that are activated in response to MAPK pathway inhibition by MEKi. Additionally, evidence of apoptosis was observed through the increased level of cleaved caspase-3 in combination-treated tumor samples, supporting the cytotoxic potential of this regimen. Therefore, dual targeting of MAPK and HSP90 disrupts primary and compensatory oncogenic pathways and can possibly achieve a more sustained tumor suppression.

The combination of SNX-2112 and selumetinib thus represents a pioneering and rational treatment approach that addresses the treatment challenges in the management of NF1-associated pNFs. Beyond HSP90i, several marketed drugs, for instance HIV protease inhibitors, have been shown to exert mild ancillary HSP90 inhibitory activity and have been repurposed in other cancers [38]. With the proof-of-principle study using potent and direct HSP90 inhibitors identified through drug mining, repurposing of those agents remains an important next step to assess their comparative effectiveness and potential synergy with selumetinib in the context of pNFs. Nevertheless, drug combination presents a method for maximizing the antitumor efficacy of the agents used to treat pNF. Importantly, the dual treatment strategy may also alleviate the therapeutic burden associated with long-term MEK inhibition. By co-targeting complementary survival mechanisms, lower doses of each agent may be sufficient to achieve desired antitumor effects, potentially reducing the incidence and severity of side effects. This dose reduction potential is particularly relevant in pediatric and adolescent patients, for whom long-term toxicity is a critical consideration. As targeted therapies continue to advance, drug combinations offer promise for personalized precision oncology, targeting resistant tumor phenotypes with reduced adverse effects. These findings underscore the therapeutic paradigm in precision medicine, which emphasizes that cancer treatments should evolve through cooperative mechanisms to dismantle multifaceted oncogenic processes. Future studies should focus on defining the molecular basis of this synergy, optimizing dose and scheduling regimens, and identifying biomarkers that may predict response to the combination. Furthermore, early-phase clinical trials could help evaluate safety and feasibility in NF1 patients, particularly in pediatric and young adult populations, where long-term tolerability is critical.

While our study provides important preclinical insights, it has certain limitations. The immortalized pNF cell lines used were established through overexpression of human telomerase (hTERT) and mouse CDK4, which, although enhancing in vitro viability and enabling long-term culture, may introduce bias in cell viability–based drug evaluation. Moreover, the in vivo drug testing was conducted using transplanted *DhhCre;Nf1^fl/fl^* tumor models, which effectively assess tumor growth inhibition but do not fully recapitulate the pNF tumorigenesis. Future studies using genetically engineered models that better mimic native tumor development will help further validate the therapeutic potential of the proposed combination strategy.

In summary, our findings support a novel combination strategy that not only improves the antitumor efficacy of selumetinib but also addresses key challenges associated with monotherapy in NF1-related pNFs. Through integrated preclinical evaluation, we demonstrate that SNX-2112 enhances the effects of selumetinib via complementary mechanisms, providing a compelling rationale for future clinical development. As the treatment of NF1-associated tumors continues to evolve, such rational combinations are likely to be at the forefront of precision medicine strategies.

## 5. Conclusions

This study highlights integrated drug mining as an effective strategy to identify therapeutic targets and combinations for pNFs. We demonstrate that combining HSP90 inhibitors, particularly SNX-2112 and retaspimycin, with the MEK inhibitor selumetinib leading to synergistic tumor growth inhibition, outperforming selumetinib alone. This combination not only enhances efficacy but may also allow for dose reduction of selumetinib, potentially minimizing associated toxicities. These findings support the clinical potential of HSP90i in treating pNFs and emphasize the importance of targeting multiple oncogenic pathways to overcome tumor heterogeneity and resistance. Further preclinical evaluations will help guide clinical translation.

## Data Availability

The original contributions presented in this study are included in the article. Further inquiries can be directed to the corresponding author.

## Abbreviations

The following abbreviations are used in this manuscript:

NF1	Neurofibromatosis type 1
pNF	Plexiform neurofibroma
HSP90i	Heat shock protein inhibitor
MPNST	Malignant peripheral nerve sheath tumor
MEKi	Mitogen-activated protein kinase inhibitor
IVIS	In vivo imaging system
